# Recruitment strategies and participant motivations in a digital randomized controlled trial for the prevention of anxiety disorders: the prevANS study

**DOI:** 10.3389/fdgth.2026.1746430

**Published:** 2026-05-13

**Authors:** Cristina García-Huércano, Sonia Conejo-Cerón, Alberto Rodríguez-Morejón, Carmela Martínez-Vispo, Natalia Sánchez-Aguadero, Olaya Tamayo-Morales, Patricia Moreno-Peral

**Affiliations:** 1 Instituto de Investigación Biomédica de Málaga y Plataforma en Nanomedicina (IBIMA Plataforma BIONAND), Málaga, Spain; 2 Department of Personality, Assessment and Psychological Treatment, University of Malaga (UMA), Málaga, Spain; 3 Research Network on Chronicity, Primary Care, Prevention and Health Promotion (RICAPPS), Barcelona, Spain; 4 Institute of Psychology (IPsiUS), University of Santiago de Compostela (USC), Santiago de Compostela, Spain; 5 Department of Clinical Psychology and Psychobiology, University of Santiago de Compostela (USC), Santiago de Compostela, Spain; 6 Unidad de Investigación en Atención Primaria de Salamanca (APISAL), Instituto de Investigación Biomédica de Salamanca (IBSAL), Salamanca, Spain; 7 Facultad de Enfermería y Fisioterapia, Universidad de Salamanca, Salamanca, Spain; 8 Facultad de Ciencias Biomédicas y de la Salud, Universidad Alfonso X El Sabio (UAX), Madrid, Spain

**Keywords:** anxiety disorders, app, digital interventions, prevention, randomized controlled trial, recruitment strategies

## Abstract

**Background:**

Anxiety disorders are among the most prevalent mental health problems worldwide, and access to effective treatment is not always available. Preventive interventions need to be scalable and cost-effective, which can be achieved through communication and information technologies. However, recruiting participants for digital prevention trials remains a major methodological challenge.

**Aim:**

To evaluate the performance of different approaches to recruiting participants for a digital preventive intervention for anxiety (the prevANS trial), and to assess participants' motivations for enrolling in the trial.

**Methods:**

Descriptive analyses were conducted to evaluate the performance of each recruitment strategy (number of potential participants attracted per week). Quantitative data were obtained from website records of individuals initiating the online screening process while each strategy was active, and self-reported information on how participants learned about the study. Baseline group differences between the intervention and control groups were examined using chi-square and Mann–Whitney *U*-tests. Reflexive inductive thematic analysis was used to analyze qualitative data on participants' main motivations for enrolling, collected through an open-ended survey question.

**Results:**

Over a 26-month recruitment period, 6,017 individuals initiated screening and 1,054 participants were enrolled (17.5% conversion rate). The most effective strategies for attracting potential participants were social media and university dissemination. Self-reported data also indicated that word of mouth had a notable impact on recruitment. The final sample was mainly composed of women and highly educated participants, and the intervention and control groups were balanced across all variables except for age. Thematic analysis revealed three main motivations for enrollment: helping others, health related issues, and own benefits.

**Conclusion:**

Recruitment strategies should be tailored to the target population, as their performance may vary across groups. Involving users through co-design and co-creation can enhance both the intervention and the identification of effective recruitment channels in digital trials.

## 1 Introduction

Anxiety disorders are characterized by the presence of severe fear, nervousness or worry along with other physiological symptoms such as shortness of breath or muscle tension whose presence causes an impairment in social, occupational or other relevant functioning areas (1). They are also among the most prevalent mental health conditions worldwide and rank as one of the leading contributors to global disease burden, affecting over 300 million people (2). Following the COVID-19 pandemic, the prevalence of anxiety symptoms has risen to approximately 33% (3). Moreover, anxiety disorders have a substantial impact in terms of disability, accounting for approximately 23% of disability-adjusted life years (DALYs), which measures the gap between the current health status of a given population and its normative life expectancy in full health, attributable to mental disorders. They are second only to depressive disorders, and they represent the eighth leading cause of years lived with disability worldwide (4). In 2020 alone, anxiety disorders were estimated to be responsible for about 44.5 million DALYs globally (ranging from 30.2 to 62.5 million) (5). This considerable clinical burden also translates into high economic impact, as individuals with anxiety disorders' healthcare costs are 2.52 times greater compared to those without these disorders (6). As for indirect costs, anxiety diagnoses are associated with both increased rates of work absenteeism and decreased levels of work performance (7). Moreover, even after remission, individuals who suffered from anxiety still show significant impairments in several areas of functioning such as interpersonal functioning, cognition or mobility (8).

Despite the availability of effective psychological and pharmacological treatments (9, 10), many individuals with anxiety disorders do not receive adequate care. On one hand, stigma still plays a relevant role when it comes to asking for help regarding mental health concerns (11). This, in addition to a general lack of information about where to get the correct assistance, constitutes an important barrier for treatment seeking (12, 13). On the other hand, diagnostic errors are a reality on daily practice, and it is common for anxiety symptoms to often be misdiagnosed as a depressive disorder, or to simply be underestimated (14–16). Lastly, financial concerns need to be noted as a clear obstacle for seeking professional help (11–13). Even when all these variables can be overcome, adherence to the interventions is usually inconsistent for many individuals (17). Moreover, the incidence of new cases is so high that, even with adequate treatments, the overall disease burden does not substantially decrease. This underscores the need to prevent these new cases of anxiety before treatment becomes necessary. However, resources for reducing incidence through prevention are still lagging behind treatment efforts, creating the so-called prevention gap (18). This makes primary prevention a key public health priority, as already recognized by the European Commission in 2013 (19). In response to this need, increasing efforts have been made to design and evaluate preventive interventions aimed at reducing the onset of anxiety disorders. Scientific evidence, including systematic reviews and meta-analysis, supports the effectiveness of psychological and psychoeducational interventions in the prevention of anxiety (20, 21). However, achieving meaningful reductions in anxiety incidence depends on ensuring that these interventions are accessible to a broad population. In this regard, it makes sense to focus on developing new approaches for anxiety prevention that are accessible and scalable, and information and communication technologies (ICTs) represent a promising solution for that matter. Digital mental health interventions, including web-based platforms and mobile applications, have emerged as promising tools for delivering scalable preventive interventions. With over 5.1 billion people using the internet globally, representing 64.4% of the world's population (22), the potential reach of digital interventions and the use of mobile devices is unprecedented. These technologies offer key advantages such as anonymous access, 24/7 availability, and flexible use. Moreover, they have the potential to reduce therapy-related costs (23) and are regarded as highly scalable and cost-effective tools for use in public health contexts (24, 25). The literature emphasizes that to achieve large scale public health impact, digital interventions must be sustainable, scalable, and adaptable (23, 26). Although evidence on digital mental health interventions for preventing anxiety disorders is still emerging (27–30), systematic reviews and meta-analyses indicate that such interventions produce small yet significant reductions in anxiety symptoms, with effect sizes ranging from 0.15 (29) to 0.31 (27, 31). More specifically, these meta-analyses report modest effect sizes for non-clinical populations (31), including small effects in young people and somewhat larger effects on individuals with subclinical anxiety symptoms (28, 29). Another analysis focusing only on adults also reports small but significant effects (27). However, some of these meta-analyses are limited by narrow age ranges, highly specific inclusion criteria (e.g., restricting analyses to self-guided programs), potential risk of bias in the included studies, and the inclusion of studies that did not exclusively target prevention. These findings suggest that the effectiveness of digital preventive interventions for anxiety disorders is still inconclusive, and the overall strength of the evidence remains very low. Therefore, more high-quality studies and interventions are needed, with larger effect sizes and improved adherence rates.

On the other hand, digital prevention trials, despite their potential scalability, face important methodological challenges, particularly regarding participant recruitment and engagement (32, 33). Recruitment strategies are a crucial yet often underestimated aspect of digital mental health research, given their direct impact on the feasibility and validity of randomized controlled trials (RCTs). These strategies are particularly critical, as they not only determine sample size and statistical power, but also shape the composition of the study population and, ultimately, the generalizability of the results (34). Online recruitment strategies have become increasingly common and offer several advantages over traditional or offline approaches. These include faster enrollment, broader geographic reach, lower costs per participant, and the ability to tailor recruitment to specific populations using digital targeting tools (35–37). Reviews and meta-analysis of online recruitment in digital health trials have highlighted these benefits, pointing to the efficiency and scalability of web-based strategies, particularly in trials requiring large sample sizes (35, 36). This is especially relevant in the field of prevention, where large samples are needed and the target population may not actively seek support (38), making online channels a valuable tool for reaching otherwise disengaged individuals. Even with all these advantages, evidence comparing online and offline recruitment methods remains mixed. While some analyses conclude that online recruitment is generally more cost-effective and faster than conventional methods (35, 36), others reveal more variable results (39). For example, in some comparisons, social media recruitment was found to be less effective or more costly than traditional methods, and only occasionally the most efficient option. Additionally, while online approaches appear notably valuable for engaging hard-to-reach or underserved populations, they do not always lead to more representative samples (39). These inconsistencies suggest that the success of online recruitment depends on factors such as population characteristics, the nature of the intervention, and the resources allocated to recruitment efforts. Despite its growing importance, few digital health trials document recruitment methods (40–42), and none focus specifically on preventive mental health interventions. Studies often fail to specify which channels were used or evaluate the effectiveness of different strategies, limiting opportunities to learn from prior efforts and to refine future trials, and ultimately leading researchers to repeatedly face the same challenges. More rigorous evaluations and transparent reporting of recruitment strategies are needed to ensure that digital preventive interventions reach the appropriate target populations efficiently and equitably (36).

Equally important is understanding why people decide to participate in digital prevention trials, as these motivations can directly influence the success of participant recruitment. While research in this area remains limited, existing studies suggest that motivations are diverse and often include altruistic reasons, such as the desire to help others or contribute to scientific advancement (43–45). Conversely, some individuals are driven by personal needs, especially during periods of emotional vulnerability or emerging symptoms, viewing the intervention as timely and relevant (43). Digital interventions also offer anonymity, flexibility, and low-threshold access to support, making them particularly attractive for individuals who may be reluctant to seek traditional in-person services. Furthermore, participants frequently report an interest in acquiring self-management skills or improving their emotional well-being, even when not clinically diagnosed (43).

While digital interventions offer a promising route to scalable and accessible mental health support, their success depends not only on the effectiveness of the intervention itself, but also on how participants are recruited and why they choose to take part. Transparent reporting of recruitment strategies and participant motivations is essential, as it enables future researchers to conduct more efficient and successful recruitment processes. In this context, the present study has two main objectives: 1) to evaluate the effectiveness of different approaches to recruiting participants for the prevANS trial; and 2) to assess participants' motivations for enrolling in the prevANS trial.

## 2 Materials and methods

### 2.1 Study design and setting

This study is part of a larger randomized controlled trial (RCT) assessing the effectiveness of the prevANS intervention, an online psychological intervention based on a risk algorithm for the prevention of anxiety disorders (46). The prevANS RCT has two arms (intervention vs. control) and three assessment periods (baseline, 6-month and 12-month follow up).

The primary outcome of the study was the cumulative 12-month incidence of any DSM-5 anxiety disorder (including panic disorder, agoraphobia, generalized anxiety disorder, or social anxiety disorder). Diagnoses were assessed through telephone interviews at baseline for participants scoring 10 points or higher on the Generalized Anxiety Disorder-7 (GAD-7) (47), and, for all participants, at 6- and 12-month follow-ups using the anxiety disorders module of the Composite International Diagnostic Interview (CIDI) (48). Further methodological details are available in the published study protocol (46).

The present study focuses on quantitative data regarding the recruitment strategies that were implemented within the prevANS RCT, and qualitative data about what motivated participants to join the trial.

Although the prevANS trial is being conducted in both Spain and Portugal, this article presents data exclusively from the Spanish sample. At the time of writing, participant screening in Portugal had not yet been completed and recruitment efforts were still ongoing, whereas in Spain recruitment has finished and follow-up assessments are currently taking place.

The trial received ethical approval from the Regional Ethics and Research Committee of Malaga. The study protocol was published in 2023 (46). The trial is registered on ClinicalTrials.gov (NCT05682365). All participants provided written informed consent prior to enrollment.

### 2.2 Elegibility and recruitment

Eligible participants were aged 18 years or older, had access to an internet-connected device, resided in Spain, and were proficient in Spanish. Individuals presenting severe mental disorders (such as psychosis, bipolar disorder, or substance use disorders), terminal illness, or cognitive impairment (e.g., dementia) were excluded. Participants were also excluded if enrolled in another research study on depression or anxiety or if they met diagnostic criteria for an anxiety disorder at baseline.

The presence of anxiety symptoms was screened using the GAD-7 (47), and participants scoring 10 or higher were further assessed with the CIDI (48) to confirm or rule out an anxiety disorder diagnosis. All trial procedures, including screening, informed consent, participant registration, randomization, intervention delivery, and data collection, were conducted entirely through a dedicated smartphone application (prevANS app), available in both Android and iOS, or web platform (https://www.prevans.org), except for anxiety diagnoses.

#### 2.2.1 Recruitment strategies

Recruitment for the prevANS RCT in Spain began on December 5, 2022 with the aim of reaching a total of 1,000 participants. Enrollment was closed on February 22, 2025, once the recruitment target was met. During the 26-month period, a broad variety of online and offline strategies were implemented:

##### 2.2.1.1 Digital press

Throughout the project, a total of twelve articles were published in various digital media newspapers across Spain, both local and national. These articles provided an overview of the objectives of the *prevANS* trial and included detailed information on how to participate through the screening website. They also outlined the inclusion criteria and presented general information about anxiety disorders, aiming to raise awareness of the issue while encouraging participation. The articles were published on December 2, 2022; January 10, 2023; September 20, 2023; September 29, 2023; October 2, 2023; October 31, 2023; November 27, 2023; December 19, 2023; March 18, 2024; March 19, 2024; April 4, 2024; and May 17, 2024. Only one article was published under payment, and the total cost was 605€.

##### 2.2.1.2 University means

Information on how to participate in the trial was also disseminated to university students. This strategy included directly informing students orally in class, sending informational e-mails, and publishing information on the university's website. This was done in eight universities across Spain: University of Malaga, University of Cadiz, University of Seville, University of Santiago de Compostela, University of Salamanca, Universidad Europea, Loyola University Andalusia, and National Distance Education University. In two of them (University of Cadiz and Loyola University Andalusia), students were offered a small grade increase in some subjects as an incentive to participate and comply with the study follow-ups. Students received this information via their professors on March 31, 2024; April 19, 2023; and May 6, 2024, via their University's website on February 6, 2023; March 7, 2023; March 14, 2023; October 10, 2023; October 30, 2023; November 23, 2023; March 10, 2024; and November 26, 2024, via their University's social media on April 18, 2023, and via their University's radio channel on November 28, 2023.

##### 2.2.1.3 Social media

We created Instagram, Facebook and X accounts for the prevANS project. In those accounts, we published content about other studies by our research team and shared whenever any articles were published in the digital press. In March 2023, we launched a paid media campaign to advertise the prevANS trial on Instagram and Facebook, as well as Google ads, for a total cost of 1000€. Ads appeared on Instagram stories and posts, as well as on Facebook, containing the links to the screening website, from March 1 to March 31, 2023. Additionally, we contacted several influencers in the mental health field and offered them the opportunity to collaborate with us, but only one of them agreed. She shared information about the study on her Instagram stories on October 6, 2023. We also created a template for sharing information through WhatsApp or e-mail. The goal was to send it to colleagues or acquaintances and ask them to share it with people who might be interested in participating. All posts emphasized the importance of preventive interventions in mental health and explained how to participate in the prevANS trial.

##### 2.2.1.4 Posters and flyers

We designed two types of posters and a tryptic flyer, and they were placed in various locations, such as health centers, hospitals, universities, libraries, and even in some companies. This specific strategy was only implemented in certain provinces: Malaga, Seville, Santiago de Compostela, and Salamanca. Both the posters and the flyers contained information about how to join prevANS. The posters also mentioned that an iPad would be raffled off to participants at the end of the study. This was used both as a recruitment and retention strategy, since participating in the giveaway required completing all three trial assessments. The total cost to print these materials was 189€.

##### 2.2.1.5 Health professionals

As some members of our research team also worked in the national health system, specifically nurses and family physicians, they recommended joining the prevANS trial to any patient who might benefit from it and who met the inclusion criteria. They also encouraged other health professionals to do the same. This was performed specifically in primary care centers across Salamanca between September 4 and September 21, 2023.

##### 2.2.1.6 TV and radio interviews

Three TV interviews and four radio interviews took place throughout the trial. The TV programs were broadcast on Malaga's local television, on October 30, 2023; May 22, 2024; and November 20, 2024. In them, different members of the prevANS team discussed the project and explained how to participate in it. Three of the radio interviews were broadcast on a national radio channel and one on a local one, on January 10, 2023; January 18, 2023; October 6, 2023; and November 22, 2023, respectively.

##### 2.2.1.7 Associations

A national LGBTIQ+ association agreed to work with our team and disseminated information about the project on their website and their social media platforms on April 13, 2023.

The previously described recruitment strategies were adapted in each city according to the resources available at the different nodes of the research group.

### 2.3 Sampling procedure

Quantitative data on recruitment strategies was obtained passively from the total sample of individuals who initiated the screening process on the project's website. Baseline information was collected from the final sample of individuals who met the inclusion criteria and completed the initial questionnaires.

Self-reported data on recruitment strategies and qualitative data on participant's motivation were obtained from a subsample of participants in the prevANS RCT. All participants were contacted via e-mail to provide this information and were subsequently reached by phone call if they had not completed the assessment. The sample for the qualitative data was considered complete when no new answers were being obtained.

### 2.4 Data collection and measurement

We maintained a record of the recruiting methods employed and the number of people who initiated the screening process each day from the beginning of the trial. As part of this assessment, all participants were invited to complete a short poll (via Google Forms), indicating that the information collected will be used for research purposes as part of the prevANS project and it is completely anonymous and confidential. The poll included the question “*How did you hear about the prevANS study?”*. This was a multiple-choice question: “through social media (Instagram, Facebook, X)”, “through the digital press (El Sur, El País, Cadena Ser, etc.)”, “through an interview on a television program (Soy Málaga, Salud Ahora)”, “through university means (by email, teaching staff, etc.)”, “through posters and/or flyers”, “through someone I know”, “through an association”, “through healthcare professionals (family physician, nurse)”, or “other” with a free text field. They were also asked “*Could you be more specific? For example, if you responded* via *social media, indicate which social network specifically (e.g., Instagram)”.*

Qualitative data comprising participants’ motivation for joining the trial was also collected in the previously mentioned poll. They were asked to describe what motivated them to participate in a free text format (*“Why did you decide to participate in prevANS?”*), with no word limit*.* This question served as a minimal topic guide, and no predefined categories or prompts were used. All information was obtained by sending an e-mail to the participants with a link to Google Forms. Participants were instructed to identify themselves using the same e-mail address used to log in to the prevANS app to ensure the authenticity of the responses. Additionally, one research collaborator made phone calls to the participants who had not completed the online poll to obtain the same information. Data collection stopped when no additional answers were received, either by Google Forms or by answering our calls. These data were accessible only to researchers and study collaborators and was secured via email verification.

In addition, eligible individuals who completed the screening process and created an account but did not log into the app or complete the baseline questionnaires were contacted by phone by a collaborator to remind them of their account and encourage their participation.

These strategies resulted in the recruitment of a sample of 1,054 participants, who were asked to complete a self-reported baseline questionnaire via the prevANS app or web, which consisted of questions on:

(a) Sociodemographic characteristics: age, sex, gender, sexual orientation, gender identity, and educational attainment.
(b) Clinical characteristics: anxious symptomatology, depressive symptomatology, probability of onset of an anxiety disorder within 12 months, and physical and mental quality of life.
(c) Psychosocial characteristics: perceived functional social support, as well as family and social stressors.

More detailed information on the measurement instruments can be found elsewhere (46).

### 2.5 Data analysis

Descriptive analyses were conducted to evaluate the performance of each recruitment strategy. For this purpose, data from the week of implementation of each strategy were analyzed. To establish a baseline recruitment flow, we calculated the overall mean number of individuals who initiated the screening process per week across the entire recruitment period, considering only those days in which no specific recruitment strategy was active. All statistical analyses were conducted using STATA statistical package (version 14.2).

Descriptive analyses were also calculated for all the variables measured in the baseline questionnaires. Differences between the intervention and control groups were examined using chi-square tests for categorical or dichotomous variables, and Mann–Whitney *U*-tests for continuous variables that did not meet the assumption of normality as assessed with the Shapiro–Wilk test. A 95% confidence interval was used for all analyses, and *p*-values < 0.05 were considered statistically significant.

The qualitative responses from the online poll were exported into an excel sheet. An inductive reflexive thematic analysis (49, 50) was then conducted by one researcher and discussed with two additional researchers to ensure the coherence and validity of the results. We followed the six steps of thematic analysis proposed by Braun and Clarke (49): (i) becoming familiar with the data, (ii) generating initial codes, (iii) identifying themes, (iv) reviewing themes, (v) defining and naming themes, and (vi) producing the report. The first step involved thoroughly reading all participant responses multiple times while taking notes on key ideas. Initial codes were then created from relevant data segments, from which preliminary themes were identified. These proposed themes were subsequently reviewed and refined collaboratively by the three researchers to ensure they accurately captured participants' motivations. Finally, themes were clearly defined and supported by representative codes, and the final report was written and reviewed by all authors of this manuscript.

## 3 Results

### 3.1 Recruitment strategies

**Figure 1:**
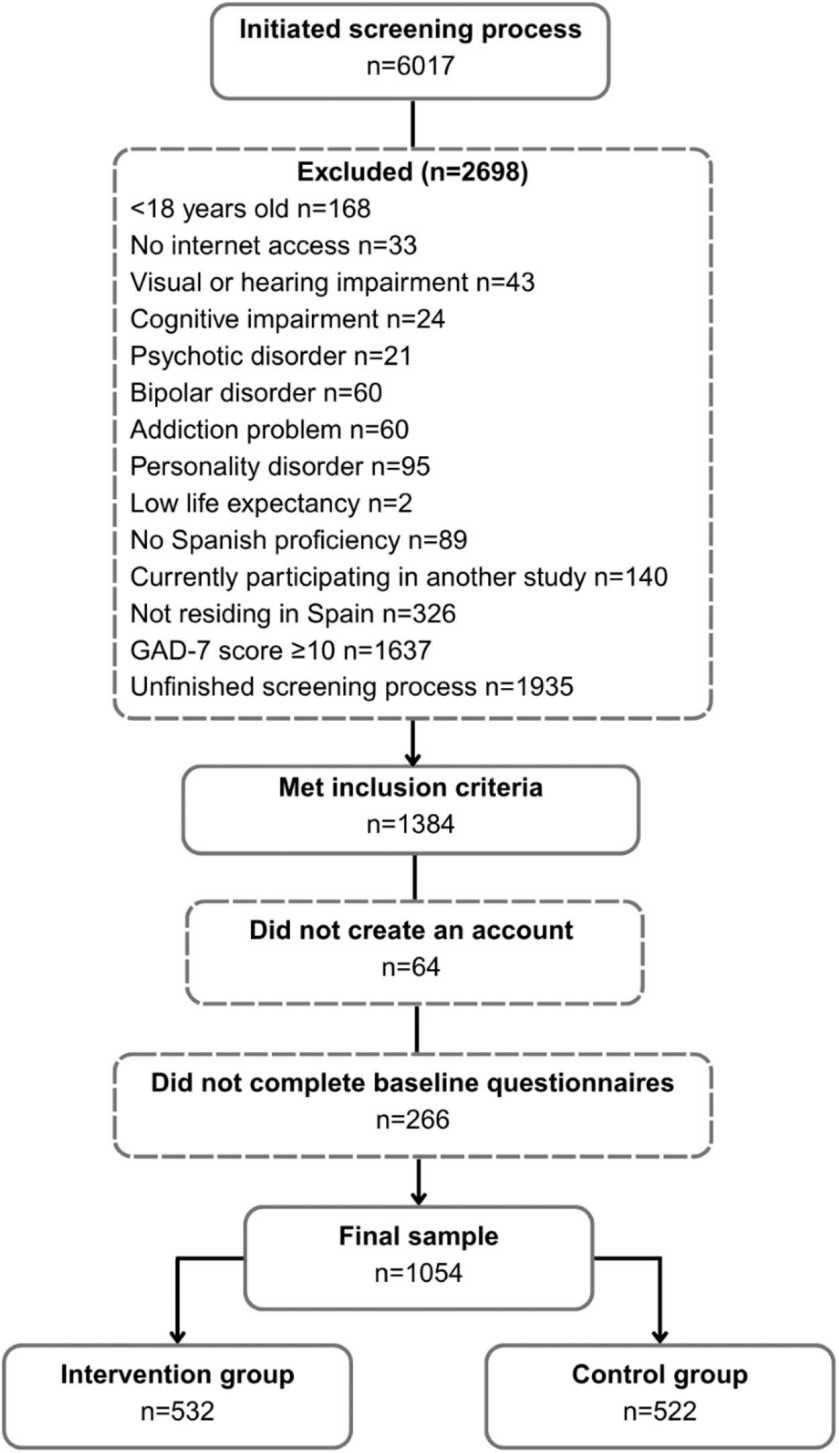
Participant flow diagram of the prevANS trial from initial screening to final sample.

A total of 6,017 individuals initiated the screening process via the prevANS website. Of these, 2,698 did not meet eligibility criteria due to reasons such as being underage, lacking internet access, having sensory or cognitive impairments, having severe psychiatric conditions (e.g., psychosis, bipolar disorder, or substance use disorders), or not residing in Spain. Additionally, 1,637 individuals scored above the clinical threshold on the GAD-7 (≥10) and either had a confirmed anxiety disorder diagnosis based on the CIDI or could not have their diagnosis confirmed because they did not complete the CIDI interview; and 1,935 did not complete the full screening process. Among the 1,384 remaining eligible individuals, 64 did not create an account and 266 did not complete the baseline assessment. The final enrolled sample comprised 1,054 participants, accounting for a conversion rate of 17.52% who were randomized into either the intervention (*n* = 532) or control group (*n* = 522). Figure 1 presents the flowchart of potential participant progression leading to the final prevANS trial sample.

Table 1 contains the mean number of participants attracted by each recruitment strategy, both individually and globally, as well as when two or more strategies were combined. On average, the most successful strategies for attracting potential participants were social media and university means, both individually and in combination.

**Table 1:** Mean number of potential participants per recruitment strategy.

Strategy	Mean (SD)	Min	Max
Digital press	69.86 (51.38)	26	166
Digital press^b^	76.92 (53.16)	26	184
Tv or radio	161.25 (124.15)	18	299
Tv or radio^b^	123.14 (100.58)	18	299
University means	44.4 (22.03)	27	82
University means^b^	184.42 (236.66)	27	648
Social media	258 (318.06)	19	619
Social media^b^	318.38 (288.72)	18	648
Associations^a^	93	93	93
Health professional^a^	11	11	11
Health professional^b^	71 (97.92)	11	184
Social media & university means	569 (96.11)	462	648
Social media & health professionals^a^	18	18	18
University means & digital press	67.33 (34.06)	28	87
University means & TV/radio^a^	82	82	82
Digital press & health professionals^a^	184	184	184
Social media, digital press & TV/radio^a^	48	48	48
University means, digital press & TV/radio^a^	87	87	87

Min, Minimum number of participants who initiated the screening process per week while the strategy was active. Max, Maximum number of participants who initiated the screening process per week while the strategy was active.

a This strategy was only active during one week-period.

b Descriptive data from each week the strategy was active, both individually and in combination.

Among the participants who constituted the final sample (*n* = 1,054), the most effective strategy globally was digital press (*n* = 281), followed by TV and radio (*n* = 240), university means (*n* = 185), social media (*n* = 81), health professionals (*n* = 44) and lastly, associations (*n* = 15).

**Figure 2:**
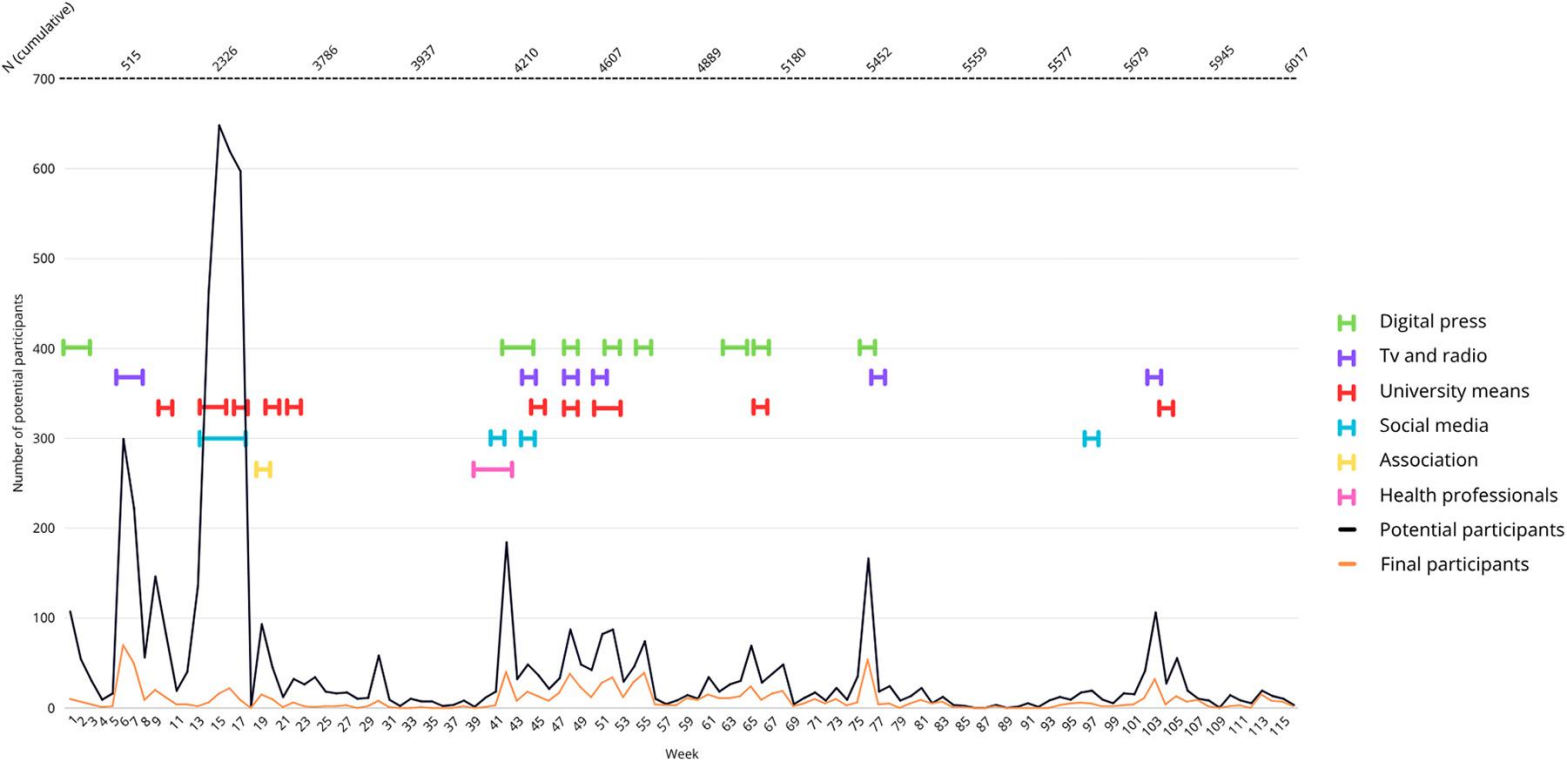
Number of potential and final participants who initiated the screening process and active recruitment strategies per week of recruitment period.

Figure 2 represents the concurrence of the different recruitment strategies carried out throughout the recruitment period, alongside the number of potential and final participants who initiated the screening process in each time period.

**Figure 3:**
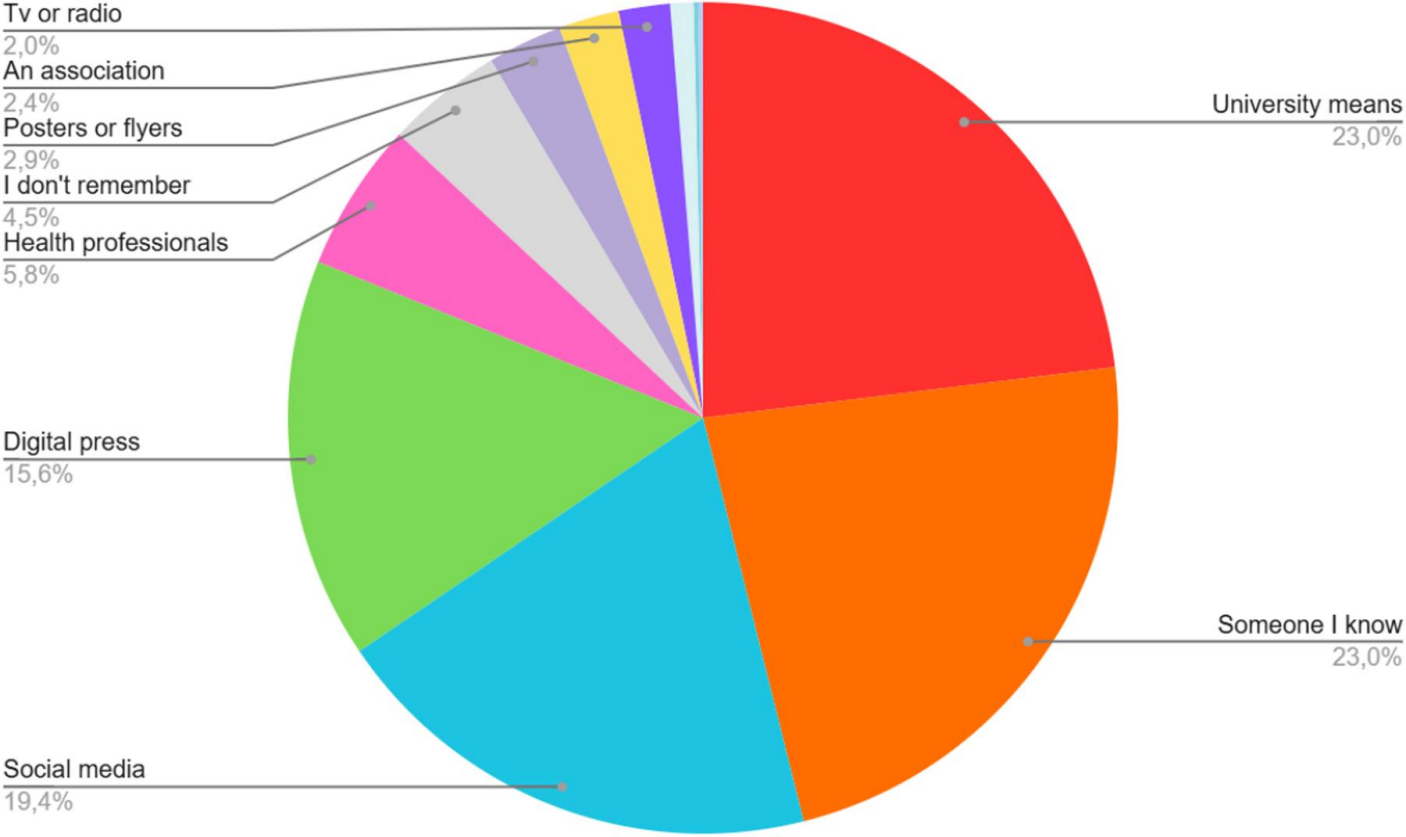
Percentage of participants who learnt about the prevANS trial through each self-reported recruitment channel.

Regarding the self-reported data, a total of 551 participants completed the poll. The strategies resulting in the highest number of final participants according to these data were university means (*n* = 127) and *word of mouth* (*n* = 127), followed by social media (*n* = 107), digital press (*n* = 86), health professionals (*n* = 32), posters or flyers (*n* = 16), associations (*n* = 13), the radio (*n* = 8), web search (*n* = 3), TV interviews (*n* = 3), job or company (*n* = 2), psychology congresses (*n* = 1), and interest in previous projects (*n* = 1). Figure 3 shows the method by which participants learned about the study, according to self-reported information.

The mean age for the whole sample (*N* = 1,054) was 37.59 years, and the majority of participants were female (70.40%), heterosexual (82.73%) and had attended university (69.07%). Both groups were balanced regarding every variable except for age, where the control group was significantly older than the intervention group (*p* = 0.015). None of the continuous variables satisfied the assumption of normality. Table 2 shows descriptive data regarding the baseline characteristics of the final Spanish sample from the prevANS trial.

**Table 2:** Baseline characteristics of the participants in the prevANS RCT (*n* = 1,054).

Variable	Intervention (*n* = 532)	Control (*n* = 522)	*p*
Mean age (SD)	36.56 (13.97)	38.64 (14.37)	0.015^f^
Sex			
Male	154 (28.95%)	150 (28.74%)	
Female	378 (71.05%)	372 (71.26%)	0.940^e^
Gender			
Man	154 (28.95%)	148 (28.35%)	
Woman	375 (70.49%)	367 (70.31%)	
Other	3 (0.56%)	2 (0.38%)	
Preferred not to answer	0 (0%)	5 (0.96%)	0.150^e^
Transsexuality			
Yes	3 (0.56%)	1 (0.19%)	
No	526 (98.87%)	516 (98.85%)	
Preferred not to answer	3 (0.56%)	5 (0.96%)	0.472^e^
Sexual orientation			
Heterosexual	436 (81.95%)	436 (83.52%)	
Homosexual	23 (4.32%)	26 (4.98%)	
Bisexual	58 (10.90%)	48 (9.20%)	
Other	4 (0.75%)	2 (0.38%)	
Preferred not to answer	11 (2.07%)	10 (1.92%)	0.782^e^
Educational attainment			
University	368 (69.17%)	360 (68.97%)	
Secondary school	148 (27.82%)	144 (27.59%)	
Primary school	15 (2.82%)	16 (3.07%)	
No studies	1 (0.19%)	2 (0.38%)	0.937^e^
Satisfaction with household coexistence			
Very satisfied	157 (29.51%)	145 (27.78%)	
Quite satisfied	227 (42.67%)	216 (41.38%)	
Neither satisfied nor unsatisfied	92 (17.29%)	94 (18.01%)	
Quite unsatisfied	47 (8.83%)	59 (11.30%)	
Very unsatisfied	9 (1.69%)	8 (1.53%)	0.718^e^
Financial strain			
Living very comfortably	131 (24.62%)	133 (25.48%)	
Living comfortably	301 (56.58%)	306 (58.62%)	
Finding it difficult	85 (15.98%)	74 (14.18%)	
Finding it very difficult	15 (2.82%)	9 (1.72%)	0.527^e^
Unpaid work			
Yes	388 (72.93%)	402 (77.01%)	
No	144 (27.07%)	120 (22.99%)	0.126^e^
Satisfaction with unpaid work^a^			
Satisfied	220 (56.70%)	231 (57.46%)	
Unsatisfied	126 (32.47%)	128 (31.84%)	
Very unsatisfied	42 (10.82%)	43 (10.70%)	0.976^e^
Mean satisfaction with unpaid work (SD)^b^^,^^a^	7.76 (3.35)	7.75 (3.26)	0.795^f^
Paid work			
Yes	374 (70.30%)	344 (65.90%)	
No	158 (29.70%)	178 (34.10%)	0.125^e^
Satisfaction with paid work^c^			
Satisfied	193 (51.60%)	176 (51.16%)	
Unsatisfied	128 (34.22%)	126 (36.63%)	
Very unsatisfied	53 (14.17%)	42 (12.21%)	0.663^e^
Mean satisfaction with paid work (SD)^b^^,^^c^	8.31 (3.54)	8.33 (3.54)	0.862^f^
Medication for anxiety/depression			
Yes	85 (15.98%)	94 (18.01%)	
No	447 (84.02%)	428 (81.99%)	0.380^e^
Alcohol/drugs abuse (relatives)			
Yes	56 (10.53%)	66 (12.64%)	
No	476 (89.47%)	456 (87.36%)	0.283^e^
Serious mental condition (relatives)			
Yes	140 (26.32%)	142 (27.20%)	
No	392 (73.68%)	380 (72.80%)	0.745^e^
Serious physical condition (relatives)			
Yes	104 (19.55%)	116 (22.22%)	
No	428 (80.45%)	406 (77.78%)	0.286^e^
Disability (relatives)			
Yes	126 (23.68%)	129 (24.71%)	
No	406 (76.32%)	393 (75.29%)	0.679^e^
Suffered physical abuse as a child			
Never	416 (78.20%)	410 (78.54%)	
Rarely	73 (13.72%)	69 (13.22%)	
Sometimes	26 (4.89%)	27 (5.17%)	
Often	12 (2.26%)	13 (2.49%)	
Very often	5 (0.94%)	3 (0.57%)	0.961^e^
DUKE-UNC-11 (item 7–8)			
Much less than I would want	21 (3.95%)	21 (4.02%)	
Less than I would want	66 (12.41%)	62 (11.88%)	
Neither much nor less	49 (9.21%)	63 (12.07%)	
Almost as much as I want	155 (29.14%)	150 (28.74%)	
As much as I want	241 (45.30%)	226 (43.30%)	0.673^e^
DUKE-UNC-11 (item 5)			
Much less than I would want	14 (2.63%)	10 (1.92%)	
Less than I would want	50 (9.40%)	47 (9.00%)	
Neither much nor less	41 (7.71%)	59 (11.30%)	
Almost as much as I want	191 (35.90%)	183 (35.06%)	
As much as I want	236 (44.36%)	223 (42.72%)	0.349^e^
Mean DUKE score (SD)	4.04 (0.99)	4.01 (0.98)	0.495^f^
Mean GAD-7 score (SD)	7.39 (3.98)	7.33 (3.98)	0.847^f^
Mean predict-A anxiety risk (SD)	14.39 (14.59)	14.73 (15.35)	0.920^f^
High anxiety risk^d^			
Yes	321 (60.34%)	321 (61.49%)	
No	211 (39.66%)	201 (38.51%)	0.701^e^
Mean PHQ-9 score (SD)	6.63 (4.32)	6.62 (4.38)	0.888^f^
PHQ-9 ≥ 10			
Yes	29 (5.45%)	32 (6.13%)	
No	503 (94.55%)	490 (93.87%)	0.637^e^
Lifetime low mood (CIDI)			
Yes	355 (66.73%)	350 (67.05%)	
No	177 (33.27%)	172 (32.95%)	0.912^e^
Lifetime anhedonia (CIDI)			
Yes	338 (63.53%)	332 (63.60%)	
No	194 (36.47%)	190 (36.40%)	0.982^e^
Mean SF-12 (Physical health) (SD)	50.60 (8.58)	50.43 (8.48)	0.637^f^
Mean SF-12 (Mental health) (SD)	43.77 (10.55)	43.90 (10.35)	0.802^f^

a N_Intervention_ = 388; N_Control_ = 402.

b Scale ranges from 4 to 23, higher scores indicate greater dissatisfaction.

c N_Intervention_ = 374; N_Control_ = 344.

d Anxiety onset probability assessed by the predict-A algorithm ≥7%.

e Derived from the chi-squared test.

f Derived from the Mann–Whitney *U*-test.

DUKE-UNC-11, Perceived Social Support Questionnaire; GAD-7, 7-item Generalized Anxiety Disorder Questionnaire; CIDI, Composite International Diagnostic Interview; PHQ-9, 9-item Patient Health Questionnaire.

### 3.2 Motivation to participate

**Figure 4:**
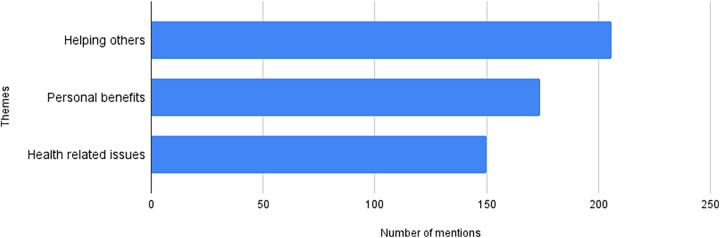
Identified themes according to motivation for participating in the prevANS trial.

A total of 546 participants responded to the poll, either online or by phone. Thematic analysis resulted in three general themes that accounted for most of the responses: helping others, health-related issues, and personal benefits. Extracts of the raw data are presented within parentheses in the descriptions of the eight identified subthemes. Figure 4 shows the number of times each theme was identified, noting that one response could contain more than one theme.

#### 3.2.1 Theme 1. Helping others

Theme 1 contains all the statements regarding participants' desire to help other people, whether by collaborating with the research project (subtheme 1.1) or by helping other people cope with anxiety (subtheme 1.2).

##### 3.2.1.1 Subtheme 1.1. Collaborating with the research project

Participants' decisions to join the prevANS study to collaborate with the project were driven by different motives, such as highlighting the importance of mental health research across society (“I understand that mental health and anything related to it is very important in the times we live in”, “Because I am concerned about mental health, both personally and collectively”, “Because research is a necessary process for scientific progress and without a sample nothing can be done”), or helping the research team (“For helping research in my field”, “Because I know how important people's participation is for research projects and because the topic itself interests me”).

##### 3.2.1.2 Subtheme 1.2. Helping other people cope with anxiety

Some participants joined prevANS to help others. Most of the time, statements referred to helping other people in general (“For helping other patients”, “…as a psychology student, I think it is something that can help many people”) but some expressed concern about helping specific people in their lives (“Because I suffer from anxiety and because I work with people with anxiety who seek my help”, “I am a nurse and I think it is a great tool to use in my practice”, “I am an educational counselor and I wanted to experiment with the tools to address anxiety problems to better help my students”, “Because I have a family member who suffers from anxiety and I thought that, by participating, I might be able to help him”).

#### 3.2.2 Theme 2. Health-related issues

Theme 2 addresses participants' concerns about both mental health (subthemes 2.1 and 2.2) and physical health (subtheme 2.3).

##### 3.2.2.1 Subtheme 2.1. Personal experience with anxiety, depression, or stress

A substantial number of participants reported experiencing past or current mental health issues, particularly anxiety (“Because I had recently suffered an anxiety crisis and wanted to tackle the problem in different ways”, “I have had problems with anxiety and agoraphobia for most of my life”), depression (“I was not feeling well, I was going through a grieving period”, “I was in a bad place trying to get out of a depressive episode”), or stress (“Because my partner and I were going through a time of great stress, anxiety and verging on depression”, “Because I “suffer” from anxiety every day and practically live in a situation of continuous stress”).

##### 3.2.2.2 Subtheme 2.2. Experience with anxiety, depression, or stress in significant others

The motivations of some participants came from experiences with mental health issues in significant others. In most cases, they referred to anxiety (“Because many in my family suffer from anxiety”, “Because I have a family member who suffers from anxiety and I thought that, by participating, I might be able to help him”, “My wife suffers from generalized anxiety and anything that can help to prevent it, if I can, I want to participate”).

##### 3.2.2.3 Subtheme 2.3. Physical health issues

Lastly, some participants mentioned that their main motivation for joining the study was related to physical health issues, in some cases leading to anxiety or stress (“When I was diagnosed with ulcerative colitis, I realized that I was living under a high degree of stress. The disease was a big blow to me and my anxiety increased”, “Because I was going through a very stressful time, moving after 25 years, complicated with ear-related problems”, “My doctor said it could be good for me”).

#### 3.2.3 Theme 3. Personal benefits

Theme 3 encompasses participants' motivations related to obtaining a benefit or a reward as a result of participating in the trial. These benefits ranged from improvements in personal health (subtheme 3.1), to satisfying their curiosity (subtheme 3.2), or winning an external prize (subtheme 3.3).

##### 3.2.3.1 Subtheme 3.1. Wanting to feel better

Several participants stated that a desire to improve their mental health or to feel better was the main reason why they joined prevANS. In this line, some people said they wanted to manage their emotions better (“To get tools to help me manage my emotions”, “I was interested in learning about exercises or ways to manage anxiety”, “To improve stress management”, “I was going through a rough patch and thought it might help me a lot”) while others put emphasis on preventing emotional problems from happening (“For working on me and understanding mental health”, “To prevent anxiety and stress-related issues”, “Because I started University this year and I think it can be useful to prevent the anxiety or stress that this change may cause me”).

##### 3.2.3.2 Subtheme 3.2. Interest or curiosity

This subtheme includes participants' desire to learn more about mental health in general, or about the prevANS project in particular, to satisfy their curiosity. In this regard, some respondents stated their profession is related to mental health (“I am a psychology student and I am interested in the subject”, “I find the subject interesting because I am a nurse and it is in the field of health”, “Because I think it could be an interesting study, I am a psychologist and I also suffer from anxiety sometimes, so I am interested in it as a professional and as a patient”), while others were simply curious about the prevANS intervention (“To know what it was about and what it could offer”, “Out of curiosity”, “Because I find it very interesting that there is an app that helps us to prevent anxiety and depression”).

##### 3.2.3.3 Subtheme 3.3. Obtaining an external prize

As noted in section 2.2.1 of this article, as part of the recruitment strategy, participants were offered two external rewards or prizes. In this case, some participants mentioned university-related benefits (“To obtain a higher score in a subject”, “I was interested in the subject matter and also participating gives us some extra points in our subjects”, “We were motivated by teachers with incentives in their subjects”) and the possibility of winning an iPad (“Because of the iPad, and because I am interested in mental health”, “I thought it was interesting and important. They were also raffling a tablet, and I thought, why not?”, “Because of the prize”).

## 4 Discussion

This study examined the performance of different recruitment strategies in a digital randomized controlled trial aimed at preventing anxiety disorders and explored the motivations driving participant enrollment. Over a 26-month recruitment period, more than six thousand individuals initiated the screening process, resulting in a final sample of 1,054 participants, which represents a conversion rate of 17.52%. The findings indicate that recruitment strategies combining social media dissemination with university-based channels, along with word of mouth, were particularly effective in attracting potential participants. In addition, thematic analysis revealed that motivations for participation were primarily driven by altruistic intentions, health-related concerns, and expectations of personal benefit.

Social media advertising seemed to be the best strategy to attract potential participants, as our campaign initially attracted nearly 3,000 individuals, but it yielded only 81 final participants. This is consistent with other studies recognizing that social media advertising is an effective tool for recruiting participants in RCTs (42, 51) evaluating online health promotion interventions, specifically through Instagram, Google Ads, and Facebook, however, conversion rates from potential to final participants are generally low (3% to 43%) (42, 51–53). We also found a significantly higher female representation in the final sample, which is in line with a recent study (51), suggesting that the Facebook algorithm favored the recruitment of women. Alongside social media, our results showed an important impact of university means for recruitment, which aligns with a recent RCT targeting university students (41) where the most effective recruitment method was direct contact via institutional email. The efficacy of each of these strategies individually likely contributed to the higher recruitment rates achieved in our study when these approaches were combined, particularly among university students, who represented the majority of our sample. Lastly, although the present study did not conduct a formal economic evaluation of recruitment strategies, some approaches required relatively limited financial investment compared to their reach. For example, the paid social media campaign and digital press dissemination were implemented with modest budgets. Future studies should examine the cost-effectiveness of recruitment channels in digital prevention trials.

The final sample showed balance between the intervention and control groups across all characteristics, except age, with the control group being older. Although the sample was not intended to be representative of the general Spanish population, it may be subject to selection bias arising from the recruitment methods employed, since the majority of participants were women, heterosexual, and highly educated. Women generally appear more likely to participate in digital prevention trials for common mental health disorders such as anxiety or depression, as demonstrated in recent RCTs (54–58). This may be due to the fact that women have a greater prevalence and risk of internalizing disorders, as well as higher rates of treatment uptake (59). Men, on the other hand, encounter some specific barriers that can hinder treatment seeking. Asking for help can sometimes lead to self-stigma, as it can be perceived as a weakness and therefore posing a threat to their masculinity (60, 61). These barriers contribute to an underrepresentation of men in mental health studies (62). Regarding educational attainment, engagement with digital interventions is usually greater among people with higher levels of education and income. Lower education, literacy, or digital skills often lead to greater barriers to accessing and engaging with digital interventions, and involving these groups in research poses additional challenges (63).

Since prevANS is a prevention trial, an important inclusion criterium was the absence of an anxiety disorder. However, the mean anxiety symptomatology scores were relatively high for the whole sample, with around 60% of participants showing a moderate-to-high risk (probability ≥7%) of developing an anxiety disorder within the next year. These findings also suggest the presence of a potential self-selection bias. Individuals experiencing higher levels of emotional distress, or those with previous experiences of anxiety, depression, or stress, may be more motivated to participate in preventive mental health programs. As a result, digital prevention trials may disproportionately attract individuals with elevated symptomatology, leading to higher exclusion rates and making it more difficult to recruit truly low-risk populations. This interpretation is supported by the thematic analysis of participants' motivations, which identified prior experiences with emotional difficulties as a key theme. In fact, one of the primary reasons for the exclusion of potential participants was failure to meet the inclusion criteria due to elevated anxiety symptom scores. These patterns highlight a key challenge in prevention research and underscore the need for more targeted recruitment strategies to effectively reach lower-risk populations.

Along these lines, the most frequently reported motivation for taking part in this preventive trial was “helping others”. This finding is consistent with previous studies showing that individuals who enroll in prevention trials are more likely to report altruistic rather than self-serving motivations, particularly in medical research (44). Many participants mentioned the importance of collaborating with research in the field of mental health, a perspective likely influenced by the fact that our final sample was composed mostly of university students, who may have a greater awareness of the value of scientific research for advancing knowledge and improving health outcomes. University students also have a high prevalence of anxiety disorders, with a median of 32% according to a recent meta-analysis, which may also explain why another identified theme was personal experience with anxiety, stress, or depression (64).

On the other hand, participation was also motivated by the possibility of personal benefit, whether this was feeling better or receiving an external prize. This has also been observed in a recent meta-analysis that showed that a small monetary incentive can significantly improve the consent and response rate of participants in RCTs (65). In our study, incentives included the chance to win an iPad, or for university students, a modest improvement in course grade.

### 4.1 Limitations

This study has some limitations that should be acknowledged. First, as mentioned above, two or more recruitment strategies overlapped in time on several occasions, complicating the interpretation of their individual effectiveness. This overlap is inherent to real-world recruitment and should be considered when interpreting the relative performance of each strategy, since potential participants could be exposed to several recruitment channels at the same time. Second, only 551 individuals who completed the screening process responded to the survey question regarding how they learned about the study. This represents approximately half of the final eligible sample of participants and excludes those who ultimately did not enroll in the trial. Similarly, the thematic analysis of participants' motivations for participation was based on 546 responses, and again, data were only available from individuals who were eligible to participate. Additionally, this information was not collected at the start of the study but rather later on. This resulted in some participants forgetting the specific reasons why they initially joined the trial. Regarding the self-reported data, although *word of mouth* was one of the most frequently reported channels, its performance could not be cross-validated with quantitative metrics of potential participants since it was not monitored by the research team. Lastly, as mentioned before, the final study sample was not representative of the Spanish population, as it was composed majorly by women, highly educated and heterosexual individuals. This limits the generalizability of the findings, since men, individuals with lower educational attainment and other minorities may be more likely to respond to other recruitment strategies, or to be driven by different motivations.

### 4.2 Implications

When considering the data as a whole, one of the main challenges in recruitment for preventive studies becomes evident: many individuals who express interest in participating often present current or past symptoms of the disorder under investigation. This leads to a considerable exclusion rate, as including them would compromise the preventive nature of the intervention and shift the focus toward treatment. In the context of the growing development of digital mental health preventive interventions, it is crucial to anticipate and address such challenges that may arise when implementing studies aimed at evaluating the effectiveness of preventive interventions.

Another noteworthy aspect is the role of word of mouth, which had a substantial, but unpredictable, impact on participant engagement. While not fully controllable, this mechanism can be encouraged through strategies such as incentivizing participants to engage others in the trial. However, such approaches must be applied with caution to avoid potential contamination between intervention and control groups.

Finally, it is important to note that recruitment strategies should always be adapted to the characteristics of the target sample. The effectiveness of each strategy will vary depending on the population of interest, and thoughtful planning is essential to reach the intended audience while maintaining the integrity of the study design. Specifically, in preventive trials, more efforts need to be made for attracting low-risk participants, other than focusing on symptom prevention. Approaches that highlight general benefits that arise from prevention, such as improving quality of life or reducing stress may be more appealing for these individuals. In this line, involving end-users through co-design and co-creation processes may not only enhance the development of the intervention itself, but also help to identify the most effective recruitment channels for assessing their effectiveness through digital trials.

## Data Availability

The raw data supporting the conclusions of this article will be made available by the authors, without undue reservation.
